# Quantitative evaluation of training method in placing miniscrews in orthodontic graduate program

**DOI:** 10.1186/s40510-022-00430-7

**Published:** 2022-10-03

**Authors:** Bobby Mitchell, Jie Liu, Sanghee Lee, Keiichiro Watanabe, Do-Gyoon Kim, Henry W. Fields, Xiaohan Guo, Lu Wei-En, Toru Deguchi

**Affiliations:** 1 College of Dentistry, The Ohio State University, Columbus, OH USA; 2 Division of Orthodontics, College of Dentistry, The Ohio State University, 4088 Postle Hall, 305 W. 12th Ave, Columbus, OH 43210 USA; 3 Department of Orthodontics and Dentofacial Orthopedics, Tokushima University Graduate School of Biomedical Sciences, Tokushima, Japan; 4 Division of Biostatistics, The Ohio State University College of Public Health, Columbus, OH USA

**Keywords:** Miniscrews, Training, Graduate program, Cone beam computed tomography

## Abstract

**Background:**

The purpose of this study was to assess the effectiveness of training residents in an orthodontic program in the placement of miniscrews by using cone beam computed tomography (CBCT) images. A total of 90 miniscrews were placed in 15 pig mandibles over a 3-year period by 15 first-year orthodontic residents. Miniscrews were divided into three groups (Control group: no radiographs; 2D group: placement with 2D radiographs; CBCT group: placement with CBCT). Proximity of the miniscrew to the neighboring root was measured. The miniscrew success rate was examined in the graduate clinic from 2015 to 2021.

**Results:**

The percentage of root contact for each group was: 36.7% (11/30), 20.0% (6/30), 0% (0/30), for the Control, 2D, and CBCT groups, respectively. The CBCT group was significantly different from the Control and 2D groups (*p* < 0.05). For root proximity, the miniscrews were significantly closer to the roots in the Control (*p* < 0.001) and 2D (*p* < 0.001) groups compared with the CBCT group. No significant difference was observed between the Control and 2D groups (*p* = 0.80). There was no significant difference among the years in the miniscrew success rate.

**Conclusions:**

Training the residents in an orthodontic graduate program using CBCT may be helpful to avoid root damage and to decrease the miniscrew failure rate.

## Background

In the past decade, miniscrews have become accepted as one of the most useful supplemental anchorage devices in orthodontics. A previous study indicated that most orthodontic residency programs (82.9%) and practitioners (69.2%) reported placing miniscrews approximately 10 years ago [1]. This has undoubtedly increased since then.

Miniscrews are frequently used by orthodontists because they can be placed without a referral, which saves time, although it is recommended that miniscrews should be placed by surgical specialists (periodontists or oral surgeons) to avoid root damage. One study indicated that 30% of miniscrews had root contact, and 20% caused root damage without the use of a surgical guide [2]. Logically, orthodontists need to be able to avoid root damage when placing miniscrews between teeth. This skill would preferably be acquired during residency training.

Past review studies have indicated that the average miniscrew failure rate is 10–20% [3, 4]. However, in our university graduate clinic, a substantially higher miniscrew failure rate was reported in the past. Moreover, there were some incidents in which miniscrews damaged the neighboring root during placement. We suggest that the use of large size miniscrews and not using cone beam computed tomography (CBCT) before miniscrew placement may have caused these incidents. Thus, we planned to perform hands-on miniscrew placement courses for the incoming residents in addition to lectures and observation of miniscrew placement to enable them to place miniscrews without contacting the roots using information obtained with and without the use of CBCT.

One known failure factor related to miniscrews is root proximity [5, 6]. It is known that the average interradicular space between the maxillary second premolar and first molar is 3.0–4.0 mm [7]. If a miniscrew with a diameter of 1.4–1.5 mm is used, there is less than 1.0 mm of space remaining on each side of the miniscrew. Given this situation, it is difficult for orthodontists to reliably and safely place miniscrews. Thus, precise analysis of the remaining space for miniscrew placement is essential.

Generally, most orthodontists do not have CBCT in their offices, so they use two-dimensional (2D) radiographs such as panoramic or periapical images to evaluate the available space. However, the distance between the patient and film [8] and the position of the patient [9] can cause image distortion in 2D radiographs, resulting in overlapping images of miniscrews and the neighboring roots that may or may not cause problems with placement. Three-dimensional (3D) analysis using CBCT provides several advantages over 2D radiographs. Past studies that investigated the accuracy of 2D panoramic [10] and periapical [6] radiographs indicated that their concordance rates with CBCT images were 41.3% and 46.5%, respectively. Panoramic radiographs also underestimate the available interradicular space for miniscrew placement [11]. Therefore, CBCT images may be required to precisely estimate the available space for miniscrew placement. This could also be a useful tool to train inexperienced orthodontists.

In this study, we hypothesized that there is no significant difference in: (1) root contact among miniscrews placed using no radiograph (Control group), 2D radiographs (2D group), or CBCTs (CBCT group) for guidance; and (2) the proximity of the miniscrew to the root between the Control group and the two experimental groups.

## Methods

To control the experience for miniscrew placement, first year orthodontic residents were selected for this study.

A total of 15 fresh (attached or disarticulated) domestic pig mandibles (*Sus scrofa*; age 6 months) were used for miniscrew insertion. This animal model is widely used for miniscrew research due to the similarity of the pig’s dentition to the human dentition, although the bone of the pig may be denser.

Prior to the actual placement, the residents had three lectures (each 90 minutes long). The first lecture included the history and basic information about miniscrews, the second was a literature review related to miniscrews, and the last lecture was based on technical information about miniscrew placement using images and videos. In addition, one lecture showed how to interpret CBCT images and how to use the software.

**Fig. 1:**
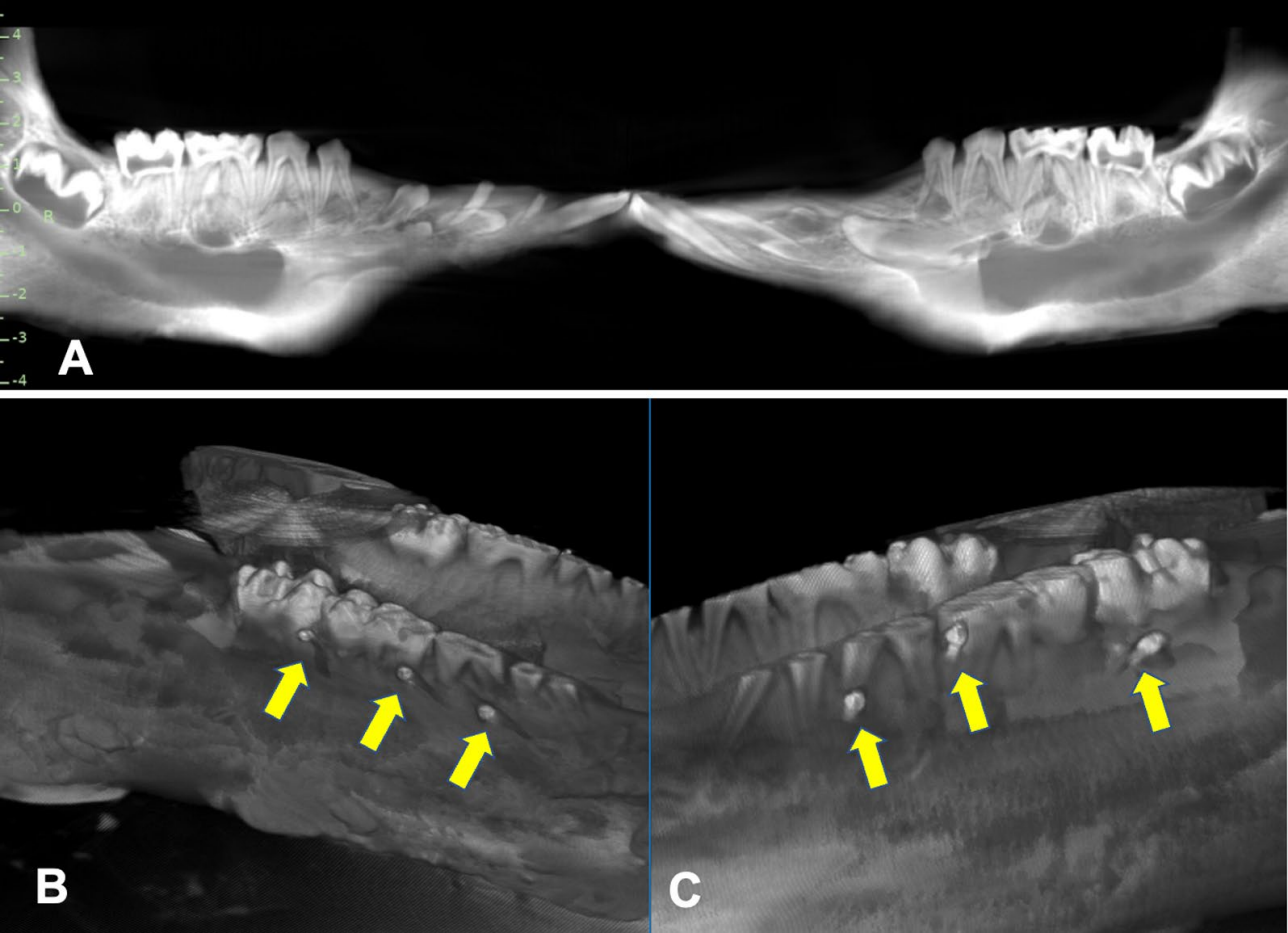
Two-dimensional radiograph **A** taken before the placement of miniscrews. Cone beam computed tomography (CBCT) radiograph (**B**: CBCT group, C: Control) after the placement of miniscrews (arrows)

All miniscrews were placed at the mesial and distal of the second molar and between the first molar and the fourth premolar. All residents were instructed to drill a pilot hole with a 1.0 mm round bur (on account of the higher bone density in pigs) and use a screwdriver to place the miniscrew. Prior to miniscrew placement, 2D radiographic images (Fig. 1A) and CBCT images were taken. A total of 90 AbsoAnchor miniscrews (1.3 mm diameter, 6 mm length, Dentos, Daegu, Korea) were placed in the interradicular regions or between the roots in the pig mandibles by 15 residents over a 3-year period (2018–2020).

Three miniscrews were placed on each side (a total of six miniscrews in each mandible) by each resident (Fig. 1B, C) using one of the following placement methods: three miniscrews on one side without any radiographic information (Control) and three miniscrews on the other side using CBCT; three miniscrews on one side without any radiographs (Control) and three miniscrews on the other side with 2D images; or three miniscrews on one side with 2D images and three miniscrews on the other side with CBCT. Therefore, a total of 30 miniscrews were installed for each of the Control, 2D, and CBCT groups. In addition, to eliminate any effect of the placement side, we placed the miniscrews for the Control, 2D, and CBCT groups equally on the right and left sides (*n* = 5 each for right and left side for all groups).

**Fig. 2:**
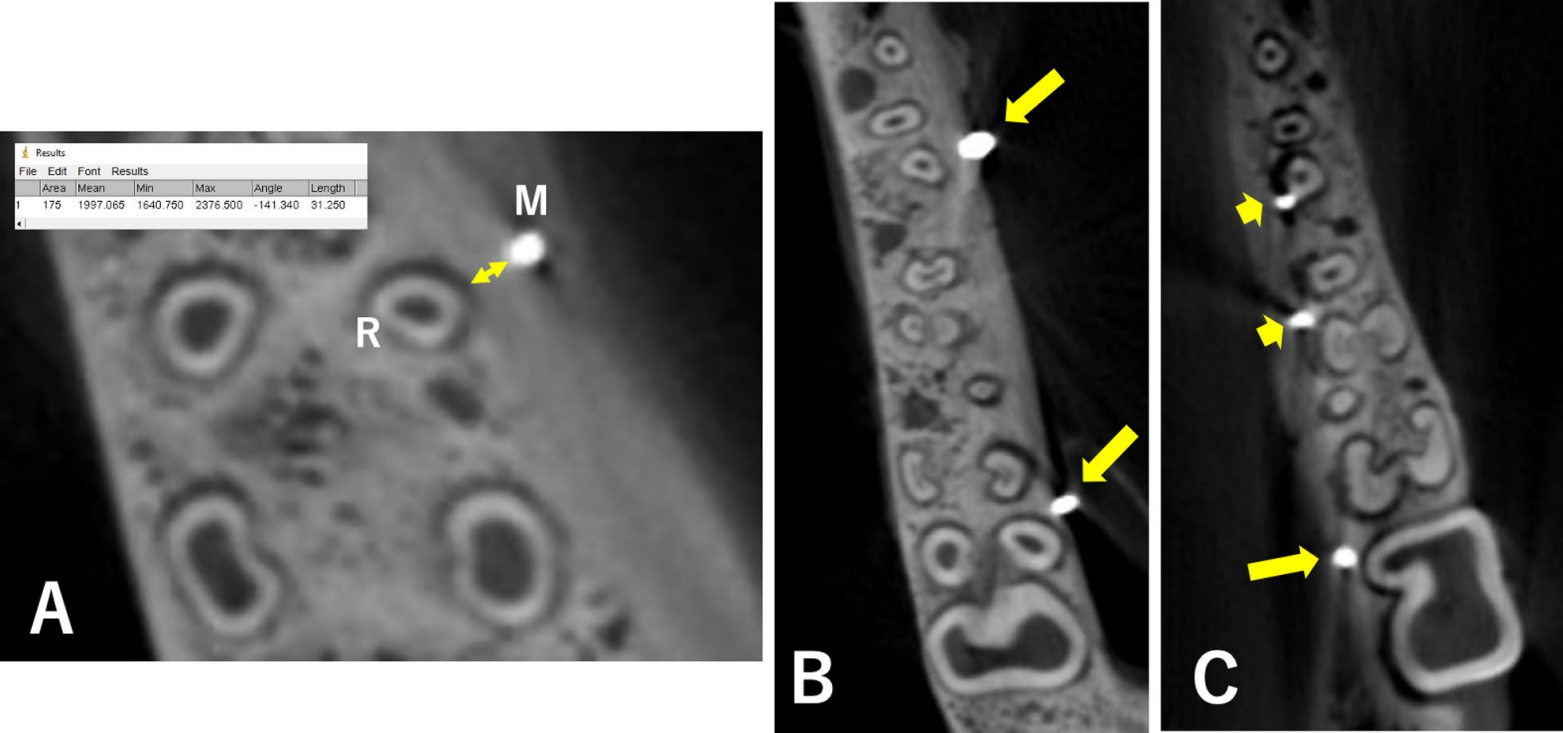
Cross-sectional cone beam computed tomography (CBCT) image showing how the distance (**A**: yellow arrow line) between the miniscrew (M) and the root (R) was measured. CBCT image showing some miniscrews were some distance from the roots (**B**, **C**: arrow), and some were close to the roots (**C**: arrowhead)

After completion of the miniscrew placement, CBCT images (Planmeca ProMax^®^ 3D Mid; Roselle, IL, USA) were taken (10 mA, 90 kV, and 200-µm voxel) to assess the root proximity (distance between the miniscrew and the closest root) of each miniscrew. We used sagittal (x-axis) and coronal (y-axis) planes to align the miniscrew with the horizontal (z-axis) plane. The z-axis was used to identify root proximity. This distance was measured using a digital ruler within Image J software (NIH, Bethesda, MD, USA) (Fig. 2A). All root proximity measurements were blinded.

The digital ruler measured pixels (equal to 0.02 mm). The miniscrew to root distance was measured at three locations: near the screw head, at the middle, and at the apex along the long axis of the miniscrew. The measured distance values were averaged for each miniscrew when the miniscrews were placed at a distance from the neighboring root (Fig. 2B). If miniscrews were shown to penetrate the root, then that distance measurement was recorded as a negative value (Fig. 2C). If the miniscrew only contacted the root, the value was 0.

In addition, we analyzed the overall success rate for miniscrews in the graduate clinic from 2015 to 2021. This was evaluated by two independent investigators. Only miniscrews that were placed in the buccal area were analyzed. Any miniscrew that was removed (or had mobility) during the treatment time was considered as a failure. It should also be noted that during 2015–2017, the same lectures (3 sessions) and a hands-on course using pig mandibles were conducted by the same instructor, but most graduate students placed only 2 miniscrews without any radiographic information.

### Statistical analysis

Root proximity was assessed by two investigators. To evaluate intraexaminer reliability, measurements for 10 randomly selected cases were repeated after at least 2-weeks. Intraexaminer and interexaminer agreement was evaluated using intraclass correlation coefficients.

Because each pig had six measurements (miniscrews), these measurements are naturally correlated. To account for this intra-animal correlation, we fitted a linear mixed effect model with the pig as the random effect and the method (Control/2D/CBCT) as the fixed effect. For each model, we conducted pairwise comparisons among the three groups with Tukey’s adjustment for multiple comparisons. The percentage of root contact was compared among the three groups by a generalized linear mixed effect model with logit link function and by Tukey’s adjustment for pairwise comparisons.

For the clinical success rate of miniscrews, comparison between the years (2015 through 2021) was performed with the chi-square test, and for comparison between 2015 to 2018 and 2019 to 2021, a two-proportions *Z* test was used.

## Results

The intraclass correlation for root proximity reliability was 0.95 (95% confidence interval: 0.81 to 0.99) (mean ± SD = −0.01 ± 0.04), and the interclass correlation was 0.71 (95% confidence interval: 0.22 to 0.92) (mean ± SD = 0.06 ± 0.17).

**Fig. 3:**
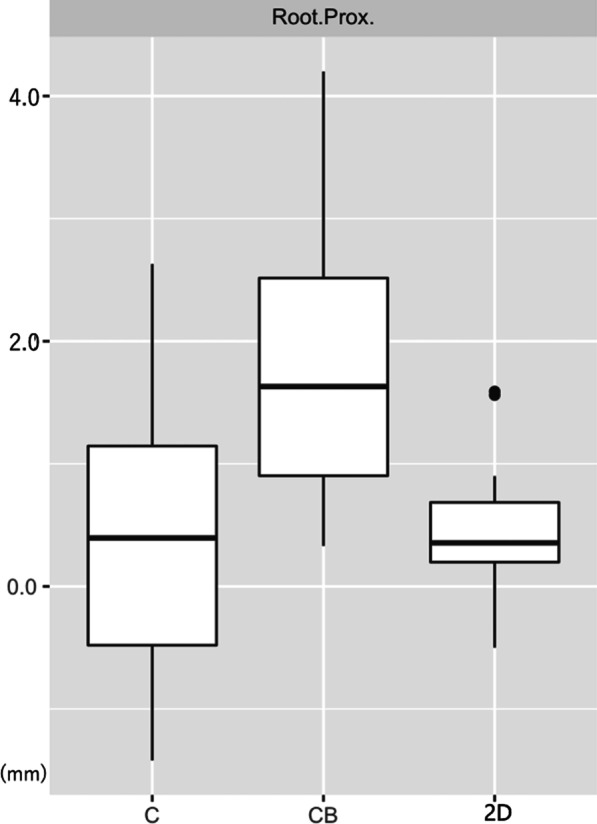
Bar graph of root proximity (Root.Prox.) between groups (C: Control, CB: CBCT, 2D: 2-dimensional radiograph)

The percentage of root contact for each group was: 36.7% (11/30), 20.0% (6/30), 0% (0/30), for the Control, 2D, and CBCT groups, respectively (Table 1) (Fig. 3). The CBCT group was significantly different from the Control group (*p* = 0.031). The 2D group was not significantly different from the CBCT (*p* = 0.13) or Control groups (*p* = 0.36).

**Table 1:** Percentage root contact with miniscrews

	Control	2D	CBCT
N sample	30	30	30
N root contact (%)	11 (36.7)^A^	6 (20.0)^A^	0 (0)^B^
Root proximity (mm) (mean ± SD)	0.04 ± 0.12^A^	0.04 ± 0.05^A^	0.17 ± 0.10^B^

^A,B^Values with similar letters are not significantly different at the *p* ≤ 0.05 level

Significantly closer root to miniscrew proximity was observed in the Control and 2D groups compared with the CBCT group (*p* < 0.001) (Table 1). No significant difference was observed between the Control and 2D groups (*p* = 0.80).

**Fig. 4:**
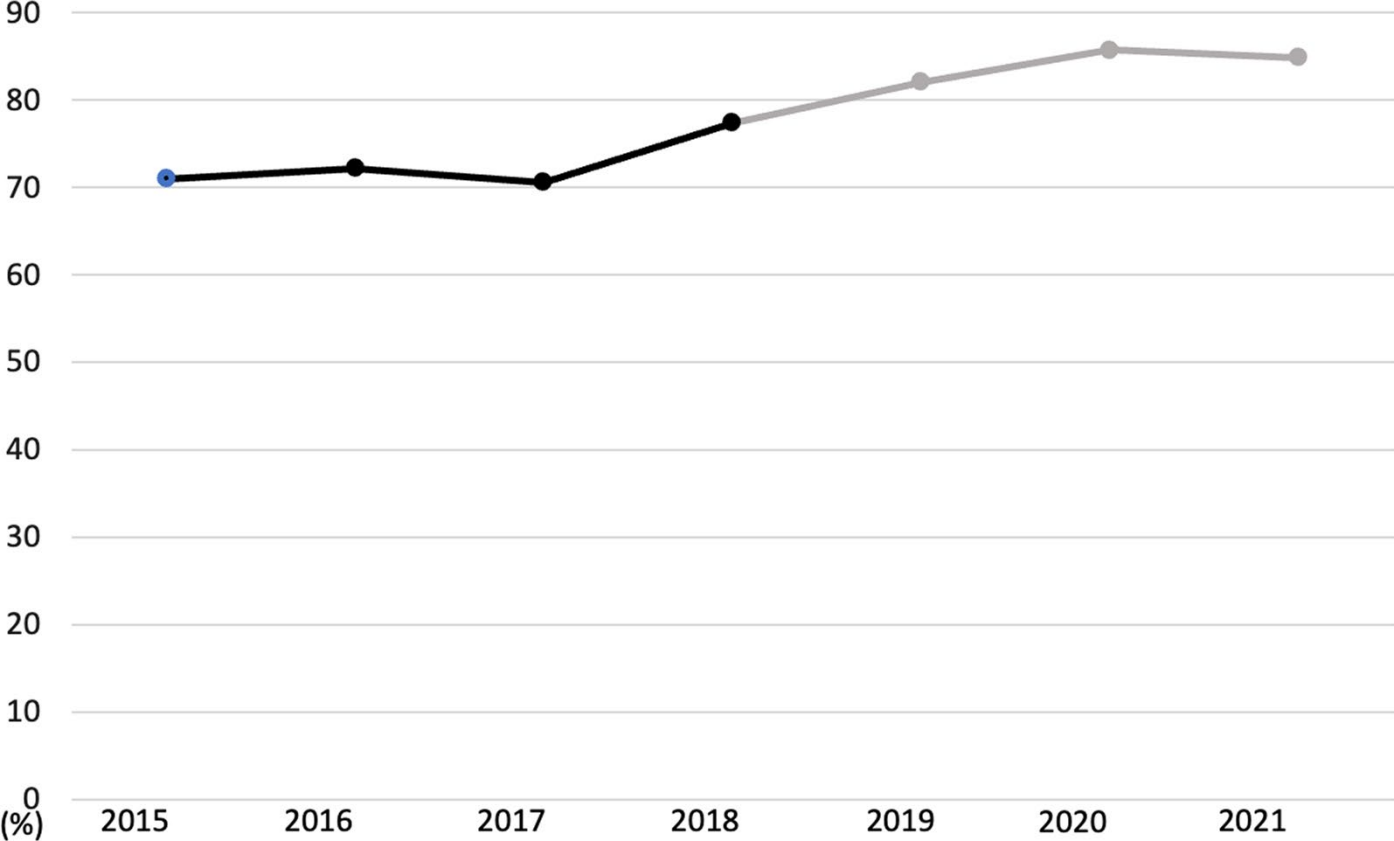
Bar graph of the success rate of miniscrews from 2015 to 2021 in the graduate clinic (dark line: before the training, gray line: after the training)

The success rate of miniscrews from year 2015 to 2021 is presented (Fig. 4). There was no significant difference among all the years in the success rate (*p* = 0.20), and also between 2015 to 2018 and 2019 to 2021 (*p* = 0.77).

## Discussion

A substantial concern is the high failure rate of miniscrews when compared with prosthetic implants [12, 13]. One previous study indicated that there was a considerable difference in the success rate of miniscrews between experienced faculty (98.1%) and orthodontic residents (70.8%) [14]. We used inexperienced residents to assess whether 2D and 3D radiographic images would affect the outcome during miniscrew placement. This process would help residents learn the steps involved in drilling and inserting miniscrews. This also put the method to its most severe test—using novice orthodontic clinicians.

One factor that has been suggested to affect the outcome of miniscrews is the placement side. Previous reviews [13, 15] have indicated that there is no significant difference in the failure rate between placement sides, but there was a tendency for the left side to result in a higher success rate. For this reason, we ensured equal placement on both sides to avoid this effect in our study.

An incorrect placement method or location can result in root damage or penetration [16], which can necessitate subsequent endodontic and prosthetic treatment.

Miniscrew failure is related to root proximity [5, 6]. When a miniscrew is placed close to a tooth root, the risk of inflammation increases, which impedes the normal healing process, resulting in decreased bone formation that may lead to poor mechanical integration between the miniscrew and the interfacial bone [17]. Thus, it is highly recommended to place the miniscrew as far as possible from the neighboring root.

In order to place miniscrews away from the root, radiographic images are essential to determine the space between the roots. There is a balance between radiographic exposure and miniscrew success combined with the type of radiographic equipment available in most orthodontic practice settings. Two-dimensional images including panoramic and periapical radiographs are commonly used in routine practice and are often included in initial records because they involve only modest radiation exposure. CBCT radiographs are less commonly available and carry more radiation burden.

In this study, placement with the use of 2D radiographic information resulted in less chance for the miniscrew to contact the neighboring roots compared with placement without any radiographic images; however, this difference was not significant. However, there was a significant difference in root contact between placement with or without CBCT images. A previous study that investigated the interradicular distance measured with CBCT and panoramic radiographs concluded that panoramic radiographs underestimated the available space [11]. Our data indicate that radiographic information, and particularly CBCT, is valuable in avoiding root contact and increasing the distance from roots. For the residents who had radiographic information from CBCT images, no miniscrews contacted the neighboring roots. Thus, for novices placing miniscrews in animal models with restricted access to interradicular bone, CBCT images were helpful in guiding the placement and avoiding root contact.

We believe it is important for residents to obtain preclinical experience with miniscrews of the type described in this study. More importantly, our findings demonstrate that in an experimentally difficult situation with novice clinicians and restricted interradicular space not subject to pre-miniscrew placement root divergence, the addition of preoperative CBCT images eliminated root contact.

The results of our clinical data of the success rate of miniscrews indicate that there were marginal differences in the success rate between the years before and after this training. The average success rate was approximately 70% before the training, increasing to approximately 85% after training. There was no significant difference between before and after the training; however, we believe that there is clinical value in increasing the success rate by approximately 15%.

It is true that placing miniscrews in pig mandibles is different from placing them in humans. Compared with the human mandible, a pig mandible has more attached gingiva and more area for placement. This could result in a lower rate of root proximity. However, there was no opportunity to diverge the roots to improve the interradicular space. In the current study, the average root proximity of the miniscrew was 1.8 mm as measured by CBCT. In comparison, a previous human clinical study [6] showed that root proximity between stable miniscrews averaged 1.7 mm. These data suggest that CBCT information is critical to achieving a root-safe and stable placement. The method was challenging because not only were the clinicians novices, they were using an animal model with human-like anatomy, but with greater restrictions on the space available for the implant insertion.

## Conclusions

The hypothesis that there was a significant difference in root proximity between the CBCT group and the Control and 2D groups was rejected. These findings suggest that with the use of CBCT, there will be less chance of root contact during miniscrew placement.

Preclinical miniscrew placement can help new residents experience common problems with miniscrew placement, with the assistance of preoperative CBCT imaging.

## Data Availability

The datasets used and/or analyzed during the current study are available from the corresponding author on reasonable request.

## Abbreviations

CBCT: Cone beam computed tomography
2D: 2-Dimensional
3D: 3-Dimensional
